# Characteristics of Serum Thyroid Hormones in Different Metabolic Phenotypes of Obesity

**DOI:** 10.3389/fendo.2020.00068

**Published:** 2020-02-28

**Authors:** Xiaomin Nie, Xiaojing Ma, Yiting Xu, Yun Shen, Yufei Wang, Yuqian Bao

**Affiliations:** ^1^Department of Endocrinology and Metabolism, Shanghai Key Laboratory of Diabetes Mellitus, Shanghai Clinical Center for Diabetes, Shanghai Jiao Tong University Affiliated Sixth People's Hospital, Shanghai, China; ^2^Shanghai Diabetes Institute, Shanghai Key Laboratory of Diabetes Mellitus, Shanghai Clinical Center for Diabetes, Shanghai Jiao Tong University Affiliated Sixth People's Hospital, Shanghai, China

**Keywords:** free triiodothyronine, free thyroxine, metabolically healthy obese, metabolically unhealthy obese, fat percentage

## Abstract

**Aim:** Metabolically healthy obese (MHO) individuals have attracted broad attention. We aimed to investigate the characteristics of serum thyroid hormones in different metabolic phenotypes of obesity.

**Methods:** The study included 1,023 community-based euthyroid subjects (age range: 27–81 years), of whom 586 were women. Fat% was detected by a bioelectrical impedance analyzer. Two definitions of obesity were applied as follows: (1) fat% ≥ 25% for men and ≥ 30% for women; (2) body mass index (BMI) ≥ 25 kg/m^2^. According to the diagnostic criteria for metabolic syndrome by the Chinese Diabetes Society, metabolically unhealthy was defined as two or more components of metabolic syndrome, excluding waist circumference. Serum-free triiodothyronine (FT3), free thyroxine (FT4), and thyroid-stimulating hormone (TSH) levels were measured by electrochemiluminescence immunoassay.

**Results:** The proportions of obesity defined by fat% and BMI were 41.3 and 27.1%, respectively. The proportion of metabolically unhealthy was 41.6%. After adjusting for age and gender, regardless of the definitions based on fat% or BMI, FT3 was positively related to both the MHO and the metabolically unhealthy obese (MUO) phenotypes [MHO: odds ratio (OR)s = 1.676 based on fat% and 2.055 based on BMI; MUO: ORs = 1.818 based on fat% and 1.526 based on BMI; all *P* < 0.05]; FT4 was negatively related to the MUO phenotype (ORs = 0.870 based on fat% and 0.849 based on BMI, all *P* < 0.05); FT3/FT4 was also positively related to both the MHO and the MUO phenotypes (MHO: ORs = 1.678 based on fat% and 2.825 based on BMI; MUO: ORs = 2.866 based on fat% and 2.883 based on BMI; all *P* < 0.05); and TSH was positively related to the metabolically unhealthy non-obese phenotype (ORs = 1.329 based on fat% and 1.321 based on BMI, all *P* < 0.01).

**Conclusions:** In euthyroid population, both the MHO and the MUO phenotypes were characterized by increased FT3 and FT3/FT4 levels.

## Introduction

Obesity is a common health problem faced by developing countries (1). Common complications of obesity include insulin resistance, dyslipidemia, hypertension, and systemic inflammation, which may finally cause type 2 diabetes, cardiovascular disease, and cancer (2–4). In recent decades, obese individuals with normal metabolic status and low risk in cardiovascular metabolic disease have gained wide attention (5). Whether the metabolically healthy obese (MHO) individuals need weight loss intervention is still unclear, and whether they can maintain stable health is also controversial (6). Clarifying these problems is essential for the targeted treatment of obesity, and may help save healthcare resources.

Thyroid hormones are closely related to both obesity and metabolic disorders (7–9). Free triiodothyronine (FT3) and thyroid-stimulating hormone (TSH) were found to be increased in morbid obesity (7). A Mendelian randomization study indicated that obesity had a causal role in increasing FT3 levels (10). The altered expression of type 1 iodothyronine deiodinase, thyroid hormone receptor, and TSH receptor in adipose tissue may partly explain the variations of thyroid hormones in obesity (11–13). Even within the euthyroid range, the change of thyroid hormones was associated with multiple metabolic risks. A prospective study found that decreased free thyroxine (FT4) within the subclinical and euthyroid range contributed to an increased risk of metabolic syndrome (MS) (8). Another cohort study found that euthyroid subjects with suspected non-alcoholic fatty liver disease had higher free triiodothyronine (FT3), lower FT4, and higher FT3/FT4 ratio (9). Higher FT3/FT4 ratio and TSH were also considered to have predictive power for MS (14). Therefore, investigating thyroid hormones in MHO individuals can help to understand and explain the potential metabolic risk of the MHO phenotype.

Although some previous studies have explored the relationships between thyroid hormones and different metabolic phenotypes of obesity, the results are quite inconsistent (15, 16). Moreover, FT3 as the bioactive form of thyroid hormones has not been analyzed in the previous studies. In the present study, we aimed to explore the relationships between thyroid hormones (FT3, FT4, and TSH) and different metabolic phenotypes of obesity in a euthyroid population.

## Subjects and Methods

### Subjects

Subjects were recruited from Shanghai communities from October 2015 to July 2016. The recruitment details are described in a previous study (17). All subjects received a complete questionnaire survey, physical examination, laboratory examination, and body composition examination. The exclusion criteria included a history of diabetes or cardiovascular diseases, abnormal thyroid function, hormone replacement treatment such as thyroid hormones or sex hormones, anti-thyroid treatment, severe hepatic or renal dysfunction, use of weight loss drugs, hypotensive drugs, lipid-regulating drugs, glucocorticoid or amiodaron, moderate to severe anemia, cancer, and acute infection. The study was approved by the Ethics Committee of the Shanghai Jiao Tong University Affiliated Sixth People's Hospital. All participants provided informed consent.

### Body Measurement and Laboratory Examination

Height, body weight, and blood pressure were measured according to standard methods described in a previous study. Body mass index (BMI) = body weight (kg)/height^2^ (m^2^). A bioelectrical impedance analyzer (TBF-418B; Tanita Corp., Tokyo, Japan) was used to detect fat%, and the details of the method are described in a previous study (17).

All subjects received a 75-g oral glucose tolerance test in the morning after an overnight fast of 10 h. Fasting plasma glucose (FPG), 2-h plasma glucose (2hPG), total cholesterol (TC), triglyceride (TG), high-density lipoprotein-cholesterol (HDL-c), low-density lipoprotein-cholesterol (LDL-c), and fasting insulin (FINS) levels were detected by methods described in the previous study (17). Homeostasis model assessment of insulin resistance (HOMA-IR) was used to evaluate the level of insulin resistance. HOMA-IR = FINS (mU/L) × FPG (mmol/L)/22.5 (18). FT3, FT4, and TSH levels were measured by electrochemiluminescence immunoassays (Roche Diagnostics GmbH, Mannheim, Germany) on a Cobas e601 analyzer, and the details of the method are described in a previous study (19).

### Definitions

According to the 1998 criteria of the World Health Organization, two definitions of obesity were applied, one was fat% ≥ 25% for men and ≥30% for women, and the other was BMI ≥ 25 kg/m^2^. According to the diagnostic criteria for MS of the Chinese Diabetes Society (CDS) (20), metabolically unhealthy was defined as having two or more of the following components of MS: (1) FPG ≥ 6.1 mmol/L or 2hPG ≥ 7.8 mmol/L; (2) systolic blood pressure (SBP) ≥ 130 mmHg or diastolic blood pressure (DBP) ≥ 85 mmHg; (3) TG ≥ 1.7 mmol/L; (4) HDL-c < 1.0 mmol/L. Waist circumference was excluded from the definitions of metabolic unhealthy due to its high multicollinearity with fat% and BMI.

According to the obesity and metabolic unhealthy status, subjects were classified into the following four different obesity metabolic phenotypes: (1) metabolically healthy and non-obese (MHNO); (2) metabolically unhealthy and non-obese (MUNO); (3) MHO; (4) metabolically unhealthy obese (MUO).

### Statistical Analysis

All statistical analyses were performed using Stata version 15.1 statistical software (StataCorp LLC, Texas, USA). The skewness/kurtosis test was used to evaluate the normal distribution of variables. All continuous variables had a skewed distribution and were expressed as medians (interquartile ranges). Categorical variables were expressed as frequencies (proportions). The Kruskal–Wallis one-way analysis of variance was used for group comparisons of skewed continuous variables. For categorical variables, the chi-square test was used for group comparisons. Multiple logistic regression analysis and marginal effects analysis were used to explore the relationships between thyroid hormones and obesity metabolic phenotypes. A two-tailed *P* < 0.05 was considered statistically significant.

## Results

### Clinical Characteristics of Subjects

**Figure 1:**
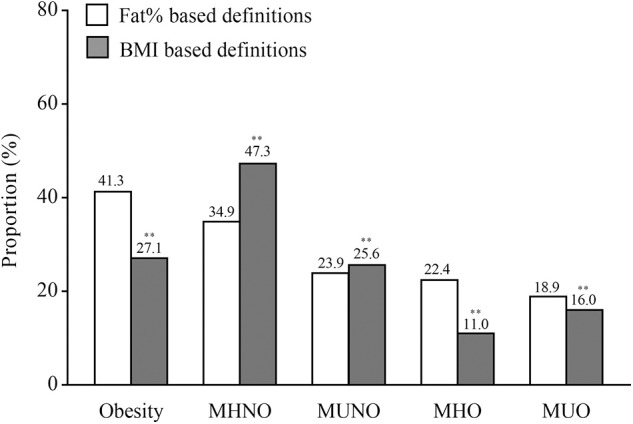
The proportions of obesity and different metabolic phenotypes of obesity. In the graph, the column indicated to proportions. For the white columns, obesity was defined as fat% ≥ 25% for men and ≥30% for women. For the gray columns, obesity was defined as BMI ≥ 25 kg/m^2^. **P < 0.01 compared with the corresponding definition based on fat%.

A total of 1,023 participants were included in the final analysis, of which 586 were women. The average age was 59 ± 8 years (age range: 27–81 years). The medians (interquartile ranges) of FT3, FT4, and TSH levels were 4.93 (4.59–5.30) pmol/L, 16.51 (15.30–17.69) pmol/L, and 2.16 (1.55–2.87) mIU/L, respectively. The proportions of obese individuals based on fat% and BMI were 41.3 and 27.1%, respectively (Figure 1). Compared with the definitions based on fat%, the proportions of the MHNO and the MUNO phenotypes were higher while the proportions of the MHO and the MUO phenotypes were lower when using the definitions based on BMI (all *P* < 0.01).

### Clinical Characteristics According to Different Obesity Metabolic Phenotypes

Regardless of using the definitions based on fat% or BMI (Table 1), compared with the MHNO subjects, both the MUNO and the MUO individuals had significantly higher BMI, SBP, DBP, FPG, 2hPG, HOMA-IR, TG, and LDL-c and lower HDL-c (all *P* < 0.05), while the MUO individuals also had significantly higher fat% (all *P* < 0.01); the MHO individuals only had significantly higher BMI, fat%, and HOMA-IR (all *P* < 0.01).

**Table 1:** Clinical characteristics according to different metabolic phenotypes of obesity.

**Parameters**	**MHNO**	**MUNO**	**MHO**	**MUO**
**Definitions based on fat%**				
*N* (men/women)	357 (180/177)	244 (202/42)^**^	229 (11/218)^**^^##^	193 (44/149)^**^^##^^ΔΔ^
Age, years	59 (54–64)	61 (55–65)^*^	58 (53–62)^##^	60 (54–64)^Δ^
BMI, kg/m^2^	21.11 (19.64–22.94)	23.03 (21.53–24.55)^**^	23.96 (22.69–25.61)^**^^##^	25.76 (23.84–27.71)^**^^##^^ΔΔ^
Fat%, %	22.10 (17.90–25.75)	20.60 (17.23–23.30)	33.30 (31.10–36.20)^**^^##^	34.20 (30.20–37.30)^**^^##^
SBP, mmHg	120 (111–129)	136 (128–148)^**^	121 (113–128)^##^	139 (131–148)^**^^ΔΔ^
DBP, mmHg	73 (68–80)	82 (76–88)^**^	73 (68–79)^##^	82 (75–89)^**^^ΔΔ^
FPG, mmol/L	5.45 (5.19–5.83)	5.91 (5.47–6.41)^**^	5.60 (5.35–5.91)^*^^##^	5.98 (5.57–6.56)^**^^ΔΔ^
2hPG, mmol/L	6.30 (5.32–7.29)	8.06 (6.75–9.44)^**^	6.56 (5.53–7.53)^##^	8.53 (7.04–10.03)^**^^ΔΔ^
HOMA-IR	1.58 (1.15–2.11)	2.19 (1.60–3.44)^**^	2.28 (1.65–3.06)^**^	3.38 (2.51–4.77)^**^^##^^ΔΔ^
TC, mmol/L	5.17 (4.56–5.78)	5.46 (4.85–6.02)^**^	5.41 (4.79–6.19)^**^	5.58 (5.04–6.29)^**^
TG, mmol/L	1.02 (0.78–1.31)	1.98 (1.34–2.73)^**^	1.14 (0.88–1.47)^##^	1.93 (1.52–2.56)^**^^ΔΔ^
HDL-c, mmol/L	1.53 (1.27–1.78)	1.23 (1.02–1.50)^**^	1.51 (1.30–1.77)^##^	1.28 (1.10–1.46)^**^^ΔΔ^
LDL-c, mmol/L	3.00 (2.53–3.60)	3.37 (2.89–3.88)^**^	3.23 (2.70–3.92)^**^	3.50 (3.07–4.10)^**^^ΔΔ^
**Definitions based on BMI**				
*N* (men/women)	484 (155/329)	262 (151/111)^**^	113 (44/69)^^##^^	164 (87/77)^**^^Δ^
Age, years	59 (54–63)	61 (56–65)^**^	58 (51–62)^##^	59 (54–64)
BMI, kg/m^2^	21.65 (20.05–23.14)	22.80 (21.67–23.79)^**^	26.14 (25.44–27.64)^**^^##^	26.62 (25.81–28.11)^**^^##^
Fat%, %	25.75 (19.32–30.70)	21.75 (17.90–30.25)	35.60 (24.25–38.30)^**^^##^	28.70 (24.23–37.98)^**^^##^
SBP, mmHg	120 (111–128)	137 (130–148)^**^	124 (114–132)^##^	140 (131–148)^**^^ΔΔ^
DBP, mmHg	73 (68–79)	82 (76–88)^**^	77 (70–81)^*^^##^	84 (75–89)^**^^ΔΔ^
FPG, mmol/L	5.47 (5.20–5.85)	5.97 (5.51–6.45)^**^	5.62 (5.43–5.87)^##^	5.96 (5.56–6.57)^**^^ΔΔ^
2hPG, mmol/L	6.41 (5.38–7.44)	8.18 (6.98–9.67)^**^	6.37 (5.49–7.60)^##^	8.49 (6.64–9.95)^**^^ΔΔ^
HOMA-IR	1.68 (1.25–2.32)	2.38 (1.70–3.54)^**^	2.48 (1.91–3.28)^**^	3.53 (2.44–4.92)^**^^##^^ΔΔ^
TC, mmol/L	5.21 (4.62–5.95)	5.60 (4.99–6.20)^**^	5.36 (4.66–5.95)^#^	5.43 (4.87–6.13)
TG, mmol/L	1.04 (0.80–1.37)	1.96 (1.30–2.80)^**^	1.19 (0.92–1.50)^##^	1.94 (1.57–2.53)^**^^ΔΔ^
HDL-c, mmol/L	1.54 (1.31–1.81)	1.28 (1.10–1.55)^**^	1.36 (1.21–1.61)^**^	1.22 (1.04–1.39)^**^^#^^ΔΔ^
LDL-c, mmol/L	3.05 (2.58–3.64)	3.48 (3.01–4.01)^**^	3.30 (2.61–3.86)	3.42 (2.94–3.93)^**^

*Data were expressed as median (interquartile range)*.

* *P < 0.05*,

** *P < 0.01, compared with MHNO*;

# *P < 0.05*,

## *P < 0.01, compared with MUNO*;

Δ *P < 0.05*,

ΔΔ *P < 0.01, compared with MHO*.

*2hPG, 2-h plasma glucose; BMI, body mass index; DBP, diastolic blood pressure; fat%, fat percentage; FPG, fasting plasma glucose; HDL-c, high-density lipoprotein; HOMA-IR, homeostasis model assessment of insulin resistance; LDL-c, low-density lipoprotein; MHNO, metabolically healthy non-obese; MUNO, metabolically unhealthy non-obese; MHO, metabolically healthy obese; MUO, metabolically unhealthy obese; SBP, systolic blood pressure; TC, total cholesterol; TG, triglyceride*.

**Figure 2:**
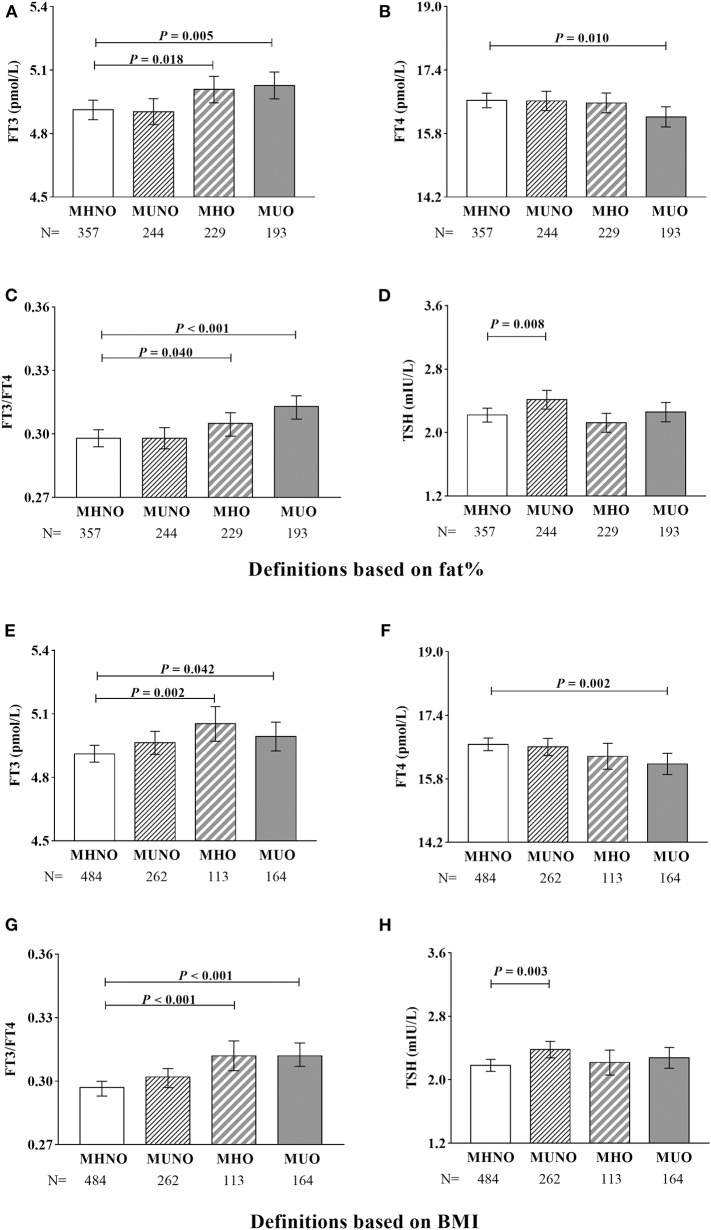
Covariance analysis of thyroid hormones in different metabolic phenotypes of obesity. **(A–D)** Definitions based on fat%. **(E–H)** Definitions based on BMI. Age and gender were adjusted. In the graph, the column indicated to adjusted mean, the bars indicated to 95% confidence interval.

Covariance analysis was further used to explore thyroid hormones levels in different obesity metabolic phenotypes after adjusting for age and gender (Figure 2). Regardless of using the definitions based on fat% or BMI, compared with the MHNO subjects, both the MHO and the MUO subjects had significantly higher FT3 and FT3/FT4 (all *P* < 0.05); the MUO subjects also had significantly lower FT4 (all *P* < 0.05); and the MUNO subjects had significantly higher TSH (all *P* < 0.01).

### Logistic Regression Analysis of Different Obesity Metabolic Phenotypes

We further used a multiple logistics regression analysis to explore the relationships between thyroid hormones and different metabolic phenotypes of obesity (Table 2). In the logistic regression analysis, age and gender were adjusted, and the MHNO subjects were set to be the reference group.

**Table 2:** Multivariate logistics regression analysis.

**Dependent variables**		**FT3**	**FT4**	**FT3/FT4^a^**	**TSH**
Definitions based on fat%	MHNO	Reference (1.000)	Reference (1.000)	Reference (1.000)	Reference (1.000)
	MUNO	0.973 (0.658, 1.439)	0.998 (0.906, 1.099)	1.037 (0.661, 1.627)	1.329 (1.081, 1.636)^**^
	MHO	1.676 (1.109, 2.535)^*^	0.983 (0.885, 1.091)	1.678 (1.032, 2.730)^*^	0.881 (0.713, 1.089)
	MUO	1.818 (1.199, 2.756)^**^	0.870 (0.783, 0.967)^*^	2.866 (1.776, 4.624)^**^	1.055 (0.853, 1.305)
Definitions based on BMI	MHNO	Reference (1.000)	Reference (1.000)	Reference (1.000)	Reference (1.000)
	MUNO	1.322 (0.927, 1.885)	0.979 (0.897, 1.069)	1.413 (0.934, 2.139)	1.321 (1.099, 1.587)^**^
	MHO	2.055 (1.294, 3.264)^**^	0.905 (0.803, 1.020)	2.825 (1.653, 4.829)^**^	1.051 (0.822, 1.343)
	MUO	1.526 (1.014, 2.297)^*^	0.849 (0.764, 0.942)^**^	2.883 (1.801, 4.615)^**^	1.142 (0.922, 1.414)

*Data were ORs (95% CI)*.

* *P < 0.05*,

** *P < 0.01*.

a *FT3/FT4 was 10 times transformed. Age and gender were adjusted*.

*BMI, body mass index; fat%, fat percentage; FT3, free triiodothyronine; FT4, free thyroxine; FT3/FT4, free triiodothyronine to free thyroxine ratio; MHNO, metabolically healthy non-obese; MUNO, metabolically unhealthy non-obese; MHO, metabolically healthy obese; MUO, metabolically unhealthy obese; TSH, thyroid-stimulating hormone*.

Regardless of using the definitions based on fat% or BMI, FT3 was significantly and positively related to both the MHO and the MUO phenotypes (all *P* < 0.05). FT4 was significantly and negatively related to the MUO phenotype (all *P* < 0.05). FT3/FT4 was also significantly and positively related to both the MHO and the MUO phenotypes (all *P* < 0.05). TSH was significantly and positively related to the MUNO phenotype (all *P* < 0.01).

**Figure 3:**
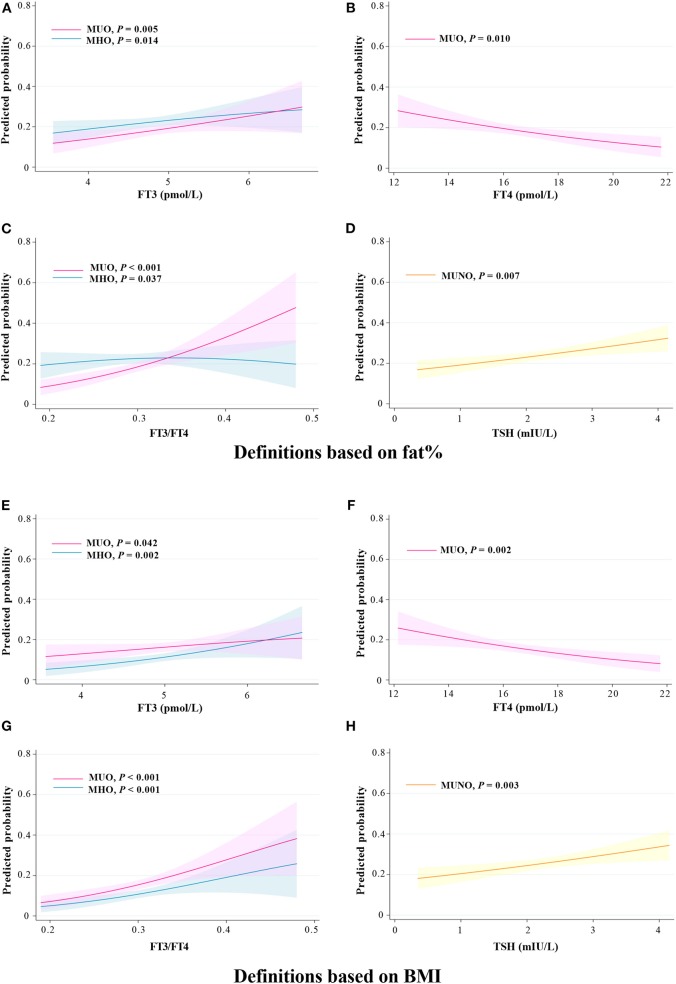
Predicted probability of different metabolic phenotypes of obesity in relation to thyroid hormones. **(A–D)** Definitions based on fat%. **(E–H)** Definitions based on BMI. Predicted probabilities and 95% confidence intervals for the marginal effect of different metabolic phenotypes of obesity.

According to the marginal effect analysis (Figure 3), regardless of using the definitions based on fat% or BMI, the probabilities of the MHO and the MUO phenotypes were significantly increased with increased FT3 and FT3/FT4 levels (all *P* < 0.05), the probabilities of the MUO phenotype were significantly decreased with increased FT4 levels (all *P* < 0.05), while the probability of the MUNO phenotype was significantly increased with increased TSH levels (all *P* < 0.01).

### Subgroup Analysis on the Gender Difference

Gender difference was further analyzed in the relationships between thyroid hormones and the obesity metabolic phenotypes (Table 3). Age was adjusted in the logistic regression analysis.

**Table 3:** Subgroup analysis on the gender difference.

**Dependent variables**		**FT3**	**FT4**	**FT3/FT4^a^**	**TSH**
**Men**					
Definitions based on fat%	MHNO	Reference (1.000)	Reference (1.000)	Reference (1.000)	Reference (1.000)
	MUNO	0.907 (0.573, 1.435)	0.938 (0.838, 1.050)	1.195 (0.711, 2.008)	1.157 (0.904, 1.481)
	MHO	1.874 (0.479, 7.337)	1.112 (0.799, 1.549)	1.186 (0.251, 5.590)	0.539 (0.228, 1.271)
	MUO	1.550 (0.727, 3.305)	0.858 (0.710, 1.038)	2.462 (1.082, 5.603)^*^	1.046 (0.694, 1.577)
Definitions based on BMI	MHNO	Reference (1.000)	Reference (1.000)	Reference (1.000)	Reference (1.000)
	MUNO	1.074 (0.644, 1.790)	0.921 (0.813, 1.044)	1.422 (0.789, 2.563)	1.371 (1.040, 1.808)^*^
	MHO	2.000 (0.921, 4.343)	0.894 (0.739, 1.082)	2.549 (1.079, 6.023)^*^	1.072 (0.704, 1.632)
	MUO	1.529 (0.831, 2.811)	0.849 (0.730, 0.988)^*^	2.696 (1.357, 5.358)^**^	0.972 (0.696, 1.357)
**Women**					
Definitions based on fat%	MHNO	Reference (1.000)	Reference (1.000)	Reference (1.000)	Reference (1.000)
	MUNO	1.016 (0.454, 2.277)	1.219 (0.999, 1.487)	0.506 (0.186, 1.371)	1.854 (1.244, 2.765)^**^
	MHO	1.683 (1.065, 2.659)^*^	0.999 (0.890, 1.123)	1.624 (0.942, 2.802)	0.956 (0.758, 1.205)
	MUO	1.842 (1.102, 3.081)^**^	0.880 (0.772, 1.003)	2.934 (1.600, 5.379)^**^	1.115 (0.863, 1.442)
Definitions based on BMI	MHNO	Reference (1.000)	Reference (1.000)	Reference (1.000)	Reference (1.000)
	MUNO	1.556 (0.939, 2.579)	1.039 (0.915, 1.180)	1.355 (0.745, 2.465)	1.248 (0.971, 1.605)
	MHO	2.067 (1.162, 3.680)^*^	0.908 (0.778, 1.059)	3.001 (1.512, 5.956)^**^	1.034 (0.764, 1.399)
	MUO	1.326 (0.742, 2.369)	0.817 (0.704, 0.949)^**^	3.106 (1.593, 6.054)^**^	1.318 (0.988, 1.758)

*Data were ORs (95% CI)*.

* *P < 0.05*,

** *P < 0.01*.

a *FT3/FT4 was 10 times transformed. Age was adjusted*.

*BMI, body mass index; fat%, fat percentage; FT3, free triiodothyronine; FT4, free thyroxine; FT3/FT4, free triiodothyronine-to-free thyroxine ratio; MHNO, metabolically healthy non-obese; MUNO, metabolically unhealthy non-obese; MHO, metabolically healthy obese; MUO, metabolically unhealthy obese; TSH, thyroid-stimulating hormone*.

When using the definitions based on fat%, FT3 was positively related to both the MHO and the MUO phenotype in women (all *P* < 0.05), but was related to none of the obesity metabolic phenotypes in men (all *P* > 0.05). In both genders, FT4 was related to none of the obesity metabolic phenotypes, while FT3/FT4 was positively related to the MUO phenotype (all *P* < 0.05). Only in women was TSH positively related to the MUNO phenotype (*P* < 0.01).

When using the definitions based on BMI, FT3 was only positively related to the MHO phenotype in women (*P* < 0.05). In both genders, FT4 was negatively related to the MUO phenotype and FT3/FT4 was positively related to the MHO and the MUO phenotypes (all *P* < 0.05). TSH was only positively related to the MUNO phenotype in men (*P* < 0.05).

## Discussion

In this study of a community-based euthyroid population, fat% measured by bioelectrical impedance analysis and BMI were used to diagnose obesity. We found that thyroid hormones were related to different metabolic phenotypes of obesity. FT3 and FT3/FT4 were all positively related to the MHO and the MUO phenotypes, FT4 was negatively related to the MUO phenotype, while TSH was positively related to the MUNO phenotype.

The major physiological functions of thyroid hormones include promoting thermogenesis and regulating energy expenditure (21). Hyperthyroidism with excess thyroid hormones is often along with weight loss, increased steatolysis and energy expenditure. The prevalence of thyroid dysfunction is not high in the general population (22); however, thyroid hormones within the euthyroid range are also found to be frequently involved in metabolic diseases. It is commonly noticed in the clinical practice that obese individuals often have higher TSH and FT3 levels (7, 23). Higher levels of FT3, FT3/FT4, and TSH were associated with increased risks of non-alcoholic fatty liver disease and MS (9, 14), while lower FT4 may contribute to an increased risk of MS (8). Whether the MHO individuals have altered thyroid hormones remains unclear.

Some previous studies have explored the relationships between thyroid hormones and obesity metabolic phenotypes. Amouzegar et al. conducted a 9-year follow-up study. They defined obesity as BMI ≥ 25 kg/m^2^ and metabolically unhealthy as three or more components of MS according to the Joint Interim Statement criteria (waist circumference was included). They found that FT4 was positively related to MHNO development and negatively related to MHO development, while TSH was positively related to MUNO development (15). In another 6-year follow-up study, Jun et al. recruited 992 euthyroid MHO individuals. They used the same criteria of obesity and metabolically unhealthy as those used in Amouzegar's study. They found that the baseline total triiodothyronine level was positively related to the risk of MS (24). In the Korean National Nutrition and Health Survey, Kim et al. also defined obesity as BMI ≥ 25 kg/m^2^. Metabolically unhealthy was defined as two or more components of MS according to the NCEP ATP III criteria (waist circumference was excluded). They found that TSH was negatively associated with MHNO status, while FT4 was positively associated with MHNO status (16).

We found large discrepancies in the proportions of obesity defined either by fat% or BMI. However, regardless of definitions based on fat% or BMI, FT3, and FT3/FT4 were all positively related to the MHO and the MUO phenotypes, among which FT4 was negatively related to the MUO phenotype, while TSH was positively related to the MUNO phenotype. Our findings in FT4 and TSH were consistent with the previous studies, suggesting that higher FT4 was related to metabolic benefits while higher TSH was related to metabolic risks. In addition, we firstly found that both the MHO and the MUO phenotypes were characterized by increased FT3 and FT3/FT4.

Gender difference was found in the relationships between thyroid hormones and the obesity metabolic phenotypes. According to the fat%-based definitions, FT3 was positively related to the MHO and the MUO phenotype in women, but not in men. Gender difference in body fat content might contribute to this discrepancy, as women tended to have higher fat% than men even with the same BMI (25). In women, FT3 was positively related to the fat%-based MUO phenotype but was not related to the BMI-based MUO phenotype. In fact, when using the BMI-based definitions, some MUO subjects moved to the MUNO group due to normal BMI despite high fat%, which might result in the non-significant result. The inconsistent results between the gender-stratified groups and the entire cohort might also be explained by the relatively small sample size in each group after gender stratifying.

The thyroid follicles secrete thyroxine and a small amount of triiodothyronine. In the peripheral tissues, thyroxine is transformed into triiodothyronine as the bioactive form by the catalysis of type 1 iodothyronine deiodinase. Triiodothyronine exerts physiological effects by binding to thyroid hormone receptor (21). The expression of type 1 iodothyronine deiodinase was enhanced in white adipose tissue of obese subjects, while the expressions of the thyroid hormone receptor TRα1 and TSH receptor were decreased (11, 12). After weight loss, the expressions of TRα1 and TSH receptor were increased, while serum FT3 and TSH levels were decreased (12). The above basic researches well explained our finding that FT3 and FT3/FT4 were positively related to obesity phenotypes independently of metabolic healthy or not. In our study, FT3/FT4 had stronger positive relationship with the MUO phenotype than the MHO phenotype, which might partly explain that FT4 was only negatively related to the MUO phenotype but not the MHO phenotype. Considering TSH was only positively associated with the MUNO phenotype, we speculated that the altered TSH levels might mainly be contributed by metabolic dysregulation, but not higher fat% or BMI. Further basic researches are needed to clarify the potential mechanisms.

Considering that higher FT3 and FT3/FT4 were related to multiple metabolic risks, our findings also indicated that the MHO phenotype may not be a benign status from an endocrine perspective. In recent years, emerging evidence has suggested that the MHO phenotype may not be a stable status. Perspective studies found that approximately half of the MHO individuals transitioned to metabolically unhealthy in the 10- or 12-year follow-up. These unstable MHO individuals also had a significantly increased risk of cardiovascular diseases (26, 27). In general, weight loss is still recommended for the MHO individuals, as obesity itself rather than metabolic status can directly affect the metabolism of thyroid hormones and then contribute to metabolic and cardiovascular risks.

Our study has some limitations. First, the causality between thyroid hormones and obesity metabolic phenotypes could not be established through a cross-sectional study. Second, the levels of thyroid antibodies and urinary iodine were not detected in our study; thus, we could not exclude potential bias from thyroid autoimmunity diseases and iodine intake. Third, the nutritional status has not been assessed in detail in this study, which might cause potential bias.

## Conclusions

In euthyroid population, both the MHO and the MUO phenotypes were characterized by increased FT3 and FT3/FT4 levels.

## Data Availability Statement

The datasets generated for this study will not be made publicly available because the ethical approval obtained for this study prevents the human data being shared publicly to protect patients' privacy. Requests to access the datasets should be directed to YB (yqbao@sjtu.edu.cn). This would be passed to the ethics committee who will decide whether they can access the data directly.

## Ethics Statement

The studies involving human participants were reviewed and approved by Ethics Committee of the Shanghai Jiao Tong University Affiliated Sixth People's Hospital. The patients/participants provided their written informed consent to participate in this study.

## Author Contributions

XM and YB designed the study. XN, YX, YS, and YW collected samples and clinical data. XN wrote the manuscript. XN, XM, and YB reviewed and edited the manuscript. All authors revised the manuscript and approved the final manuscript.

### Conflict of Interest

The authors declare that the research was conducted in the absence of any commercial or financial relationships that could be construed as a potential conflict of interest.
